# An *ex vivo* method to evaluate vasoactivity induced by hemoglobin-based oxygen carriers in resistance vessels

**DOI:** 10.3389/fbioe.2024.1376806

**Published:** 2024-06-28

**Authors:** Hang Yu, Daoyuan Gao, Guoxing You, Weidan Li, Ying Wang, Yuzhi Chen, Lian Zhao

**Affiliations:** Academy of Military Medical Sciences, Academy of Military Science of the Chinese People’s Liberation Army, Beijing, China

**Keywords:** vasoactivity evaluation, resistance artery, noradrenaline, hemoglobin-based oxygen carriers, nitric oxide scavenging

## Abstract

Red blood cell substitutes offer a solution to the problem of blood shortage and side effects of blood transfusion. Hemoglobin-based oxygen carriers (HBOCs) are one of the promising substitutes for red blood cells. Vasoactivity, which refers to the side effect of HBOCs that causes vasoconstriction and subsequent hypertension, limits the clinical application of HBOCs. In this study, an *ex vivo* method for the evaluation of vasoactivity induced by HBOCs was established based on isolated rat mesenteric artery vessels and the DMT120CP system. The DMT120CP system, equipped with a flowmeter, permits the control of intravascular pressure, pressure gradient, and flow conditions with high accuracy, simulating the physiological conditions for isolated vessels. The concentration of noradrenaline was optimized to 1 × 10^−6^∼3 × 10^−6^ M. PEGylated bovine hemoglobin (PEG-bHb) was synthesized and perfused into the vessel for vasoactivity evaluation, with bHb as the positive control and PSS buffer solution as the negative control. PEG-bHb showed a hydration diameter of 15.5 ± 1.4 nm and a P_50_ value of 6.99 mmHg. PEG-bHb exhibited a colloid osmotic pressure of 64.1 mmHg and a viscosity of 1.73 cp at 40 mg/mL. The established vasoactivity evaluation method showed significant differences in samples (bHb or PEG-bHb) with different vasoactivity properties. The vasoconstriction percentage induced by PEG-bHb samples synthesized in different batches showed coefficients of variation less than 5%, indicating good applicability and repeatability. The established evaluation method can be applied to study the vasoactivity induction and elimination strategies, promoting the clinical application of HBOCs.

## 1 Introduction

The development of alternatives to red blood cells (RBCs) is expected to solve the problems of blood shortage and potential adverse effects of blood transfusion. Hemoglobin (Hb)-based oxygen carriers (HBOCs) have been developed as alternatives to RBCs with oxygen-carrying function. HBOCs are synthesized through cross-linking, microencapsulation, or chemical modification based on Hb (Ye et al., 2023). PEGylation, a conjugation of polyethylene glycol (PEG) chains, can effectively increase the circulation time of Hb and avoid the nephrotoxicity of Hb by increasing its hydrodynamic volume. PEGylation of Hb can also significantly increase the solution properties of Hb, including colloidal osmotic pressure and viscosity. Due to the improved solution properties, the PEGylated Hb can function as a plasma expander to restore blood volume and recover micro-vascular compensation. These unique properties have also been attributed to the molecular basis of the PEGylated Hb to neutralize the vasoactivity of Hb, a major obstacle for the therapeutic application of Hb. Thus, some PEGylated Hb products (e.g., Euro-Hb and MP4) have been developed as HBOCs for clinical trials (Wang et al., 2017). HBOCs possess advantages including the absence of cross-matching, elimination of the risk of disease transmission, and easy storage and transportation. HBOCs are promising candidates for providing oxygen therapeutics, particularly in the battlefield and other critical areas where the demand for blood transfusions is high (Uter et al., 2021).

As alternatives for blood transfusion, there are still some limitations for HBOCs that need to be addressed, which limits their clinical application. One of the primary issues is vasoactivity, which is the side effect of HBOCs, that causes vasoconstriction and subsequent hypertension. The data from a meta-analysis show that the incidence of hypertension observed after infusion of HBOCs was about two times higher than that of the control group (Natanson et al., 2008). Two hypotheses have been proposed to describe the mechanisms underlying HBOC-induced vasoactivity. One hypothesis is that HBOCs scavenge nitric oxide (NO), which is produced by vascular endothelial cells and plays a crucial role as a vasodilator. Free Hb and small-sized HBOC molecules can penetrate the gap between endothelial cells and bind to NO, leading to vasoconstriction (Riess, 2001; Portoro et al., 2020; Song et al., 2020). Another hypothesis is related to the oversupply of oxygen, which is caused by HBOCs with low oxygen affinity. These HBOCs lead to the early release of oxygen before reaching the anoxic tissue, increasing the oxygen supply to the local arterial wall, and consequently causing reflex vasoconstriction of arterioles (Winslow, 2003; Song et al., 2020). Vasoconstriction caused by HBOCs can lead to hypertension and hypoperfusion (Cabrales et al., 2012). Therefore, the evaluation of vasoactivity induced by HBOCs is essential in the design and development phase.

At present, *in vivo* and *ex vivo* methods are used to evaluate the vasoactivity induced by HBOCs. For *in vivo* evaluation*,* an HBOC sample was injected intravenously, and vasoactivity was reflected through arterial blood pressure (Vandegriff et al., 2003). Compared with the *in vivo* method, *ex vivo* methods require fewer experimental animals and samples. *Ex vivo* methods based on isolated vessels have been reported in the literature. For instance, Xiong Y. et al. isolated afferent arterioles attached to glomeruli and perfused through a set of perfusion pipets handmade from glass tubes. The tension of afferent arterioles was reflected through the diameter changes after the perfusion of Hb-based microparticles (Xiong et al., 2013). The perfusion pipets need to be made by hand, which makes it difficult for the popularization of the evaluation method. Kim H.W et al. isolated rodent pulmonary artery and aortic rings and then secured the vessel ring to a glass holder with a stainless hook. The changes in vascular tension were observed during the perfusion of Hb or the vasodilator (Kim and Greenburg, 1995). In addition to afferent arterioles and large arteries, resistance vessels are rich in smooth muscles and play an important role in regulating systemic vascular resistance (De Mello, 2016; Brito et al., 2017; Graton et al., 2019). This study aims to establish an *ex vivo* method for evaluating HBOC-induced vasoactivity based on diameter changes in resistance vessels. The DMT120CP system, a commercial device, was employed to simulate the physiological conditions for isolated vessels. The DMT120CP system is equipped with a flowmeter to permit the control of intravascular pressure, pressure gradient, and flow conditions with high accuracy. This makes it possible to conduct *ex vivo* studies of blood vessel segments under conditions very close to those of *in vivo* studies.

According to the characteristics of HBOC samples, the DMT120CP system was optimized to establish an evaluation method of vasoactivity induced by HBOCs. Furthermore, PEGylated bovine Hb (PEG-bHb) was applied for vasoactivity evaluation for applicability and repeatability verification. The method described in this study may provide an easy way to evaluate HBOC-induced vasoactivity *ex vivo* and help establish a foundation for promoting the clinical applications of HBOCs.

## 2 Materials and methods

### 2.1 Animals

Male SD rats (3∼8 weeks, 220∼260 g) were purchased from Charles River Biotechnology (Beijing). All operations involving animal experiments were approved by the Experimental Animal Ethics Committee of the Military Medical Research Institute.

### 2.2 Buffer solution formula

Pysiological salt solution buffer solution: 129.99 mM NaCl, 4.69 mM KCl, 1.18 mM KH_2_PO_4_, 1.18 mM MgSO_4_·7H_2_O, 14.88 mM NaHCO_3_, 5.55 mM glucose, 0.029 mM EDTA, and 1.60 mM CaCl_2_.

KPSS buffer solution: 74.76 mM NaCl, 59.95 mM KCl, 1.18 mM KH_2_PO_4_, 1.18 mM MgSO_4_·7H_2_O, 14.88 mM NaHCO_3_, 5.55 mM glucose, 0.029 mM EDTA, and 1.60 mM CaCl_2_.

Optimization of DMT120CP system applied for HBOCs-induced vasoactivity evaluation.

**FIGURE 1:**
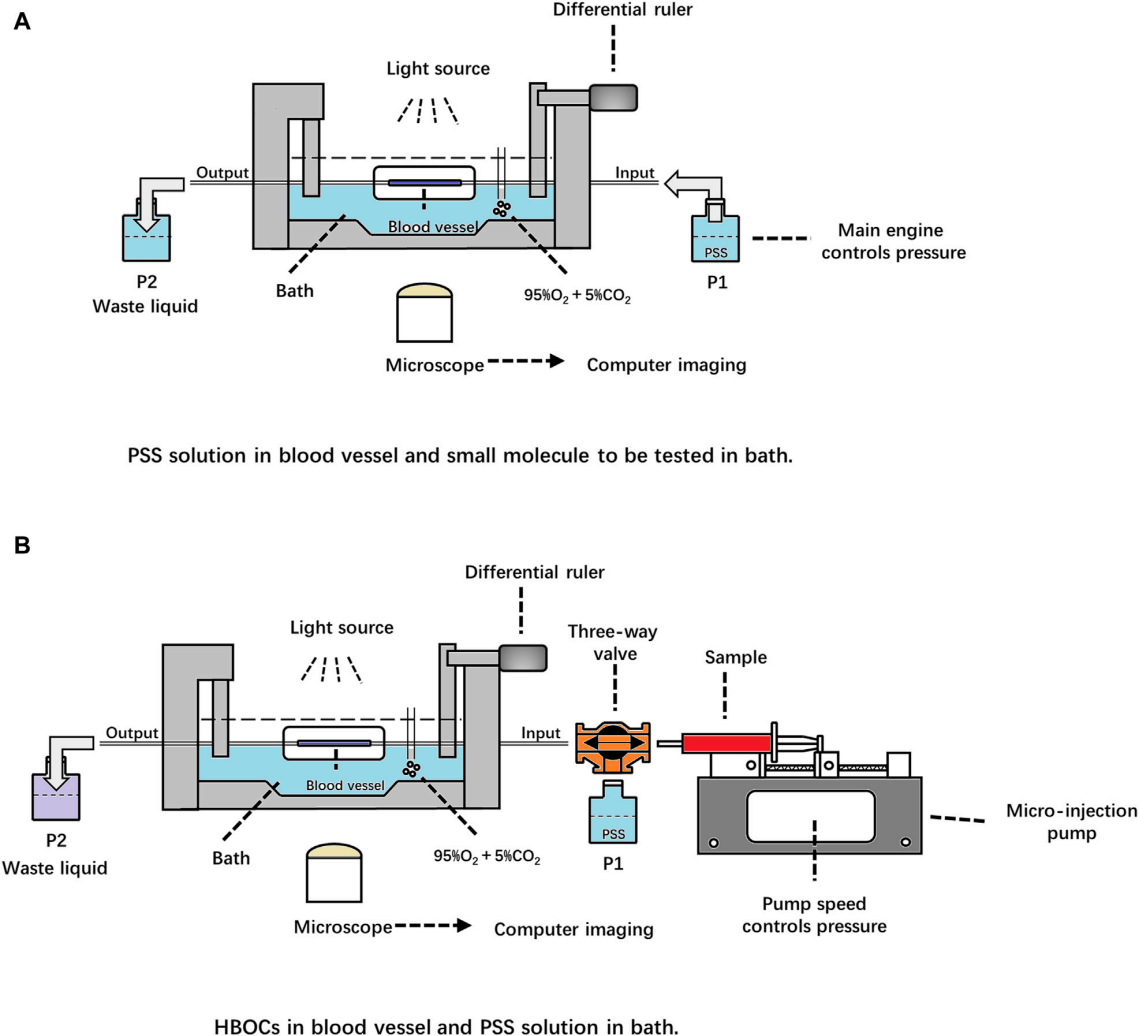
Schematic diagrams of the DMT120CP system applied for HBOC-induced vasoactivity evaluation before **(A)** and after **(B)** optimization.

Figure 1A shows the schematic diagram of the original microvascular pressure–diameter perfusion detection system (DMT120CP, DMT, Denmark). The sample of small molecules was added to the bath, and the PSS solution was driven by the pressure difference to flow through the vessel. The dilation or constriction of the vessel was observed using an inverted microscope. To evaluate the HBOC-induced vasoactivity, a three-way valve device was added to allow the exchange of the perfusate between the PSS and HBOCs, as shown in Figure 1B. The micro-injection pump was used to drive the flow of the perfusate in the vessel, while the pressure at both ends of the vessel was controlled by adjusting the pump speed.

### 2.3 Preparation of the mesenteric artery vessel *ex vivo*

The male SD rats were euthanized, and then the intestinal tissue was quickly removed and stored in PSS buffer solution (4°C) with gas bubbling (95% O_2_ and 5% CO_2_). The oxygen concentration was adjusted according to the instruction provided by DMT. A section of the intestinal tissue was cut off and fixed in a black Petri dish. The connective tissue and adipose tissue around the mesenteric artery were stripped under an inverted microscope. The second- and third-order branches of the mesenteric artery with a diameter of 100∼300 μm (Westcott and Segal, 2013) were selected, and 2 mm of the intact vessel with no branches or lacerations was cut. The two ends of the vessel were fixed on the glass tube of the inlet and outlet ends in the bath with a nylon rope (Enouri et al., 2011). To simulate the physiological state, the PSS buffer solution was added to the bath with gas bubbling (95% O_2_ and 5% CO_2_) (Trautner et al., 2006; Enouri et al., 2011; Tian et al., 2012), and the bath temperature was set to 37°C. The PSS buffer solution was perfused through the vessel by adjusting the pressure of P1 and P2. The initial pressure values of both the inlet and outlet ends were adjusted to 10 mmHg. The pressure was increased at a gradient of 10 mmHg per 5 min until it reached 60 mmHg, and then the vessel was allowed to reach equilibrium for 60 min.

After reaching the equilibrium, the PSS buffer solution in the bath was replaced with the KPSS solution, which induced vasoconstriction. After 10 min, the KPSS solution in the bath was replaced with the PSS buffer solution, leading to the recovery of vascular diameter. This process was repeated twice.

### 2.4 Preparation of PEG-bHb

PEG-bHb was synthesized according to the methods reported in the previous literature (Vandegriff et al., 2003; Wang et al., 2017). Bovine Hb (bHb), 2-iminothiolane (2-IT), and mPEG-maleimide (mPEG-Mal) were dispersed in the PBS buffer solution at a molar ratio of 1:10:20. The reaction system was stirred at 4°C for 14 h. The reaction mixture was then purified using an ultrafiltration centrifuge tube with a cutoff molecular weight of 50 kDa to remove the unreacted mPEG-Mal and bHb. The purified PEG-bHb was stored at −80°C for further use. For the synthesis of PEG-CObHb, carbon monoxide (CO) gas was vented into the PEG-bHb solution.

### 2.5 Characterization of PEG-bHb

Size exclusion chromatography (SEC): The SEC analysis was performed using a Superdex 200 column and protein purification system (Pure 25L, AKTA, Sweden). The chromatographic column was fully equilibrated and eluted with 10 mM phosphate buffer (pH 7.0) at a flow rate of 0.05 mL/min. The elution curve was recorded at 280 nm to obtain the desired data.

Oxygen affinity: The sample was dispersed to a buffer solution with an Hb concentration of 0.75 mg/mL. The oxygen dissociation curve was recorded using an Hb oxygen-binding–dissociation analyzer (BLOODOX-2018, Softron, China) at 37°C. The P_50_ (partial pressure of oxygen when Hb oxygen saturation is 50%) value was obtained from the oxygen dissociation curve (Wang et al., 2017).

Colloidal osmotic pressure (COP): The COP of the samples was measured using a Colloid osmometer (OSMOMAT^®^ 050, Gonotec, Germany). The sample with an Hb concentration of 40 mg/mL in PBS (pH 7.4) was injected into the measuring cell, and then the instrument began to measure automatically. Each sample was measured three times (Wang et al., 2017).

Viscosity: The viscosity of the samples was measured by using a rheometer (ViscoQC 300L, Brookfield Engineering, United States) with a shear rate of 75 s^-1^ at 37°C. The sample had an Hb concentration of 40 mg/mL in PBS. Each sample was measured three times (Wang et al., 2017).

Molecular particle size: The hydration diameter of particles was measured by using a Nano-ZS Particle Size Analyzer (Malvern, United Kingdom). The Hb concentration of samples was adjusted to 0.4 mg/mL in PBS. Each sample was measured three times (Wang et al., 2017).

Fourier-transform infrared (FTIR) spectrum: The FTIR spectrum was recorded by using the Thermo Scientific Nicolet iS20 spectrometer. Spectral data were processed using the Bruker software system Opus 7.2 (Bruker, Germany) (Deygen and Kudryashova, 2016).

Nuclear magnetic resonance (NMR) spectrum: The NMR spectrum was recorded by using the Bruker AV 600-MHz NMR spectrometer with CDCl_3_ as the solvent and tetramethylsilane (TMS) as the internal standard (Alvares et al., 2016).

### 2.6 Evaluation of PEG-bHb-induced vasoactivity

The sample of PEG-bHb, with a bHb concentration of 40 mg/mL in PBS, was perfused through the vessel using a microinjection pump and the three-way valve connection. The inlet pressure was maintained at 60–65 mmHg. After the sample was perfused for 15 min, noradrenaline (NE) solution was added to the bath. As the concentration of NE increased gradually from 1 × 10^−6^ to 3 × 10^−6^ M, the vessel diameter was recorded. At the end of the measurement, the solution in the bath was replaced with KPSS solution (10 mL) to induce vasoconstriction. The vasoconstriction percentage was calculated and compared with that in the vessel preparation stage. If the difference in the vasoconstriction amplitude was less than 10%, it was concluded that the experimental data were reliable and effective. In addition, the perfusion of bHb into the vessel was considered the positive control group, while the PSS solution was considered the negative control group. After the experiments, the vessels were collected, and hematoxylin and eosin (H&E) staining was performed. The vasoactivity of PEG-CObHb was evaluated with the same method.

## 3 Statistical analysis

The experimental data were presented as mean ± SEM. Normality and homogeneity of variance tests were conducted to validate the data. IBM SPSS Statistics 26 was utilized for statistical analysis. ANOVA was employed to identify differences between various concentrations of NE. One-way independent ANOVA was performed to compare different groups. Statistical significance was determined by *p < 0.05 and **p < 0.01.

## 4 Results

### 4.1 Preparation of mesenteric arterial vessels

**FIGURE 2:**
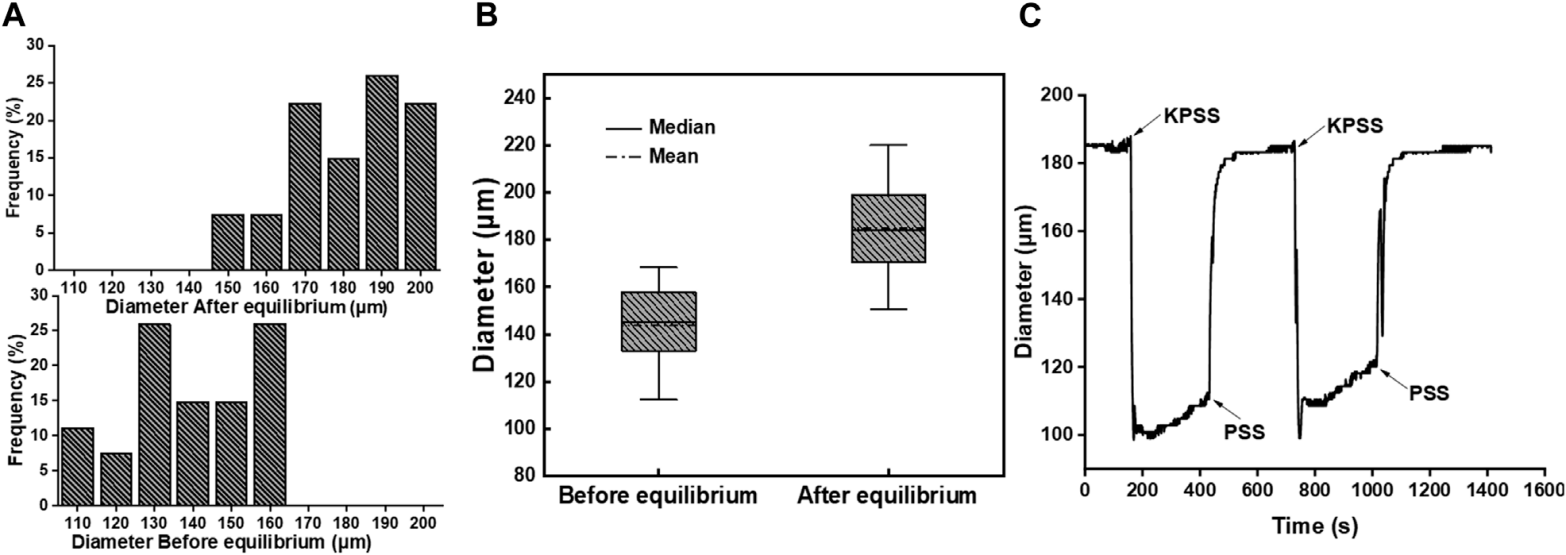
**(A)** Diameter distribution frequency of mesenteric arterial vessels before and after equilibrium (*n* = 27). **(B)** Quartile diagram of vascular diameter distribution. **(C)** Changes in the vascular diameter with buffer solution replacement between PSS and KPSS in the bath.

The diameter of mesenteric arterial vessels was measured before and after the equilibration of the PSS buffer solution. The distribution of the vascular diameter is presented in Figure 2A. The vascular diameter before equilibrium ranged from 110 μm to 160 μm. To simulate physiological conditions, the vessel was perfused with the PSS buffer solution, and the pressure at both ends of the vessel was adjusted to 60 mmHg. After reaching equilibrium, the vascular diameter increased significantly compared to that at the resting state, ranging from 150 μm to 200 μm. Figure 2B shows the quartile diagram of vascular diameter distribution, depicting the corresponding minimum, 25%, median, 75%, maximum, and average diameters. The values were 112.4 μm, 134.1 μm, 145.0 μm, 156.5 μm, 168.4 μm, and 143.8 μm, respectively, before equilibrium, and 150.7 μm, 170.9 μm, 184.2 μm, 197.8 μm, 220.0 μm, and 184.9 μm, respectively, after equilibrium.

After equilibration, the vasoconstriction of the vessel was stimulated with the KPSS buffer solution. As shown in Figure 2C, the vascular diameter decreased rapidly after the buffer solution in the bath was replaced from PSS to KPSS. The vascular diameter decreased from 185 μm to approximately 110 μm, with a vasoconstriction percentage of 40.54%. Following the removal of KPSS instead with PSS, the vascular diameter returned to 185 μm. The operation was repeated twice, and the vasoconstriction percentage was approximately 35.13% in the second time. The difference in the contraction amplitude of the vascular diameter was less than 10%, which indicated that the endothelium integrity of the vessel was still maintained, and the vessel could be used for the next vasoactivity evaluation (Trautner et al., 2006).

### 4.2 Application of NE as a vasoactivity magnifier

**FIGURE 3:**
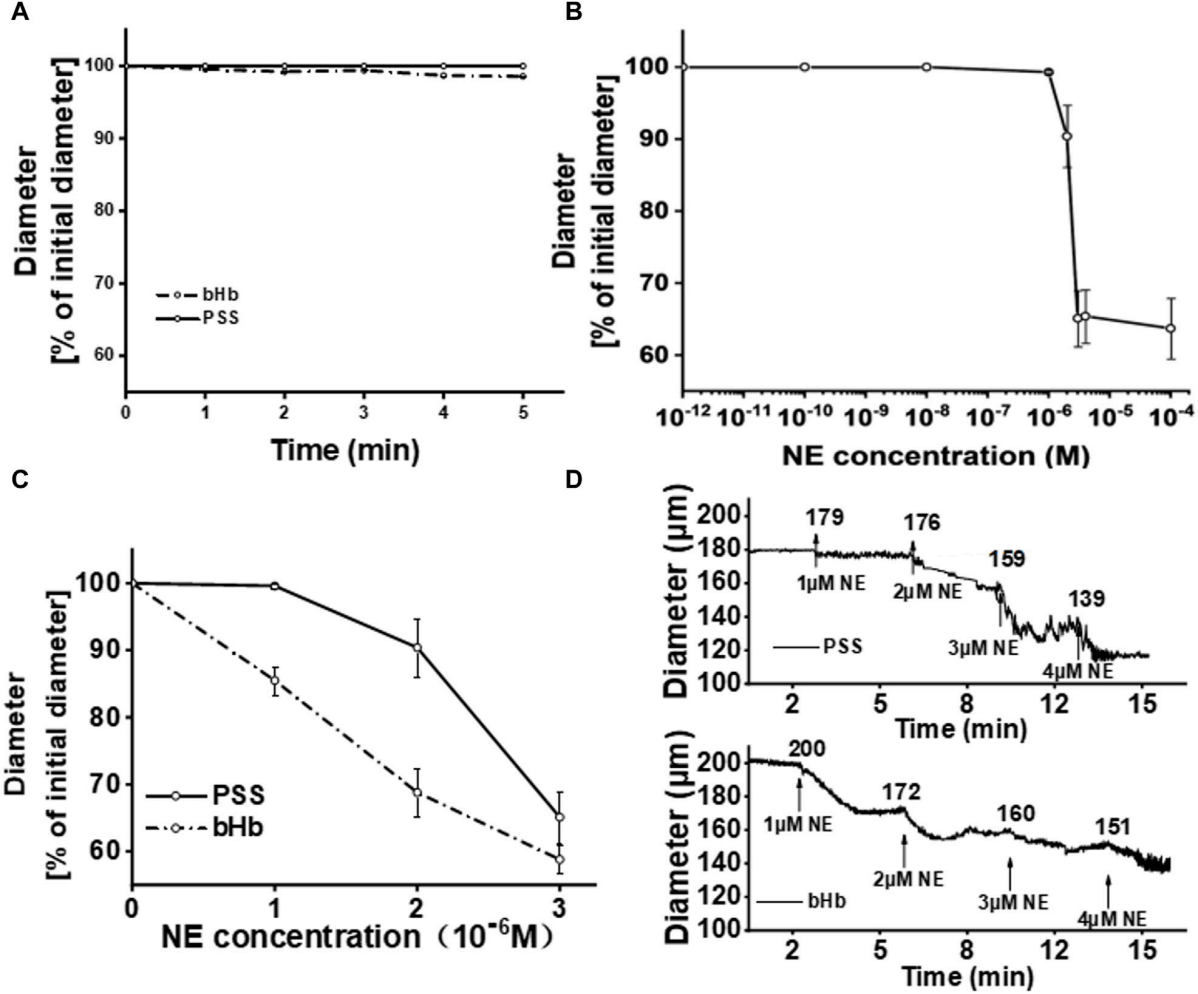
**(A)** Percentage of the initial diameter from vessels perfused with PSS buffer solution or bHb, respectively. The diameters of vessels after the perfusion of PSS buffer solution for equilibration were chosen as the initial diameters. **(B)** Percentage of the initial diameter from vessels with the increase in the NE concentration in the bath. **(C)** Percentage of the initial diameter from vessels perfused with PSS buffer solution or bHb in the presence of NE (1 × 10^−6^∼3 × 10^−6^ M) in the bath. **(D)** Diameter of vessels perfused with PSS buffer solution and bHb, respectively.

For the establishment of the vasoactivity evaluation method, the perfusion of bHb was considered the positive control group, while PSS buffer solution was considered the negative control group. The vascular diameter after the perfusion of bHb or PSS buffer solution was recorded, and the results are shown in Figure 3A. The vascular diameter remained unchanged when the vessel was perfused with the PSS solution. After perfusion of bHb, the vessel constricted slightly with a vasoconstriction percentage of 3%. To magnify the changes in the vascular diameter for vasoactivity evaluation, the NE solution was added into the bath. As shown in Figure 3B, the vascular diameter remained unchanged when the NE concentration was in the range 1 × 10^−12^ to 1 × 10^−8^ M. As the concentration of NE increased to 1 × 10^−6^ M, the vessel constricted with a vasoconstriction percentage of 0.7%. When the concentration of NE increased from 2 × 10^−6^ M to 4 × 10^−6^ M, the vessel constricted with a vasoconstriction percentage of 9.6%, 34.9%, and 34.6%, gradually. As the concentration of NE increased to 1 × 10^−4^ M, the vessel constricted significantly with a vasoconstriction percentage of 36.3%. As the concentration of NE further increased to 1 × 10^−2^ M, the vasoconstriction percentage was similar to that observed at an NE concentration of 1 × 10^−4^ M, and the vessel constriction threshold was reached. Therefore, the concentration of NE was optimized from 1 × 10^−6^ M to 3 × 10^−6^ M for vasoactivity evaluation. As shown in Figure 3C, when the concentration of NE increased from 1 × 10^−6^ M to 3 × 10^−6^ M, the vasoconstriction percentage of the vessel perfused with the PSS buffer solution was 0.6%, 8.7%, and 31.3%, while the vasoconstriction percentage of the vessel perfused with bHb was 14.5%, 31.2%, and 41.2%. NE magnified the changes in the vascular diameter for vasoactivity evaluation. As shown in Figure 3D, the vascular diameter was recorded during vasoactivity evaluation. After equilibrium, the sample was perfused into the vessel, and then NE with a gradient concentration of 1 × 10^−6^ M∼4 × 10^−6^ M was added to the bath. When the concentration of NE in the bath was 1 × 10^−6^ M, the diameter of vessels perfused with bHb showed a decrease from 200 μm to 172 μm, while the PSS buffer solution group showed a slight reduction in diameter of vessels from 179 μm to 176 μm. With the increase in the NE concentration, the amplitude of vasoconstriction increased.

### 4.3 Characterization of PEG-bHb

**FIGURE 4:**
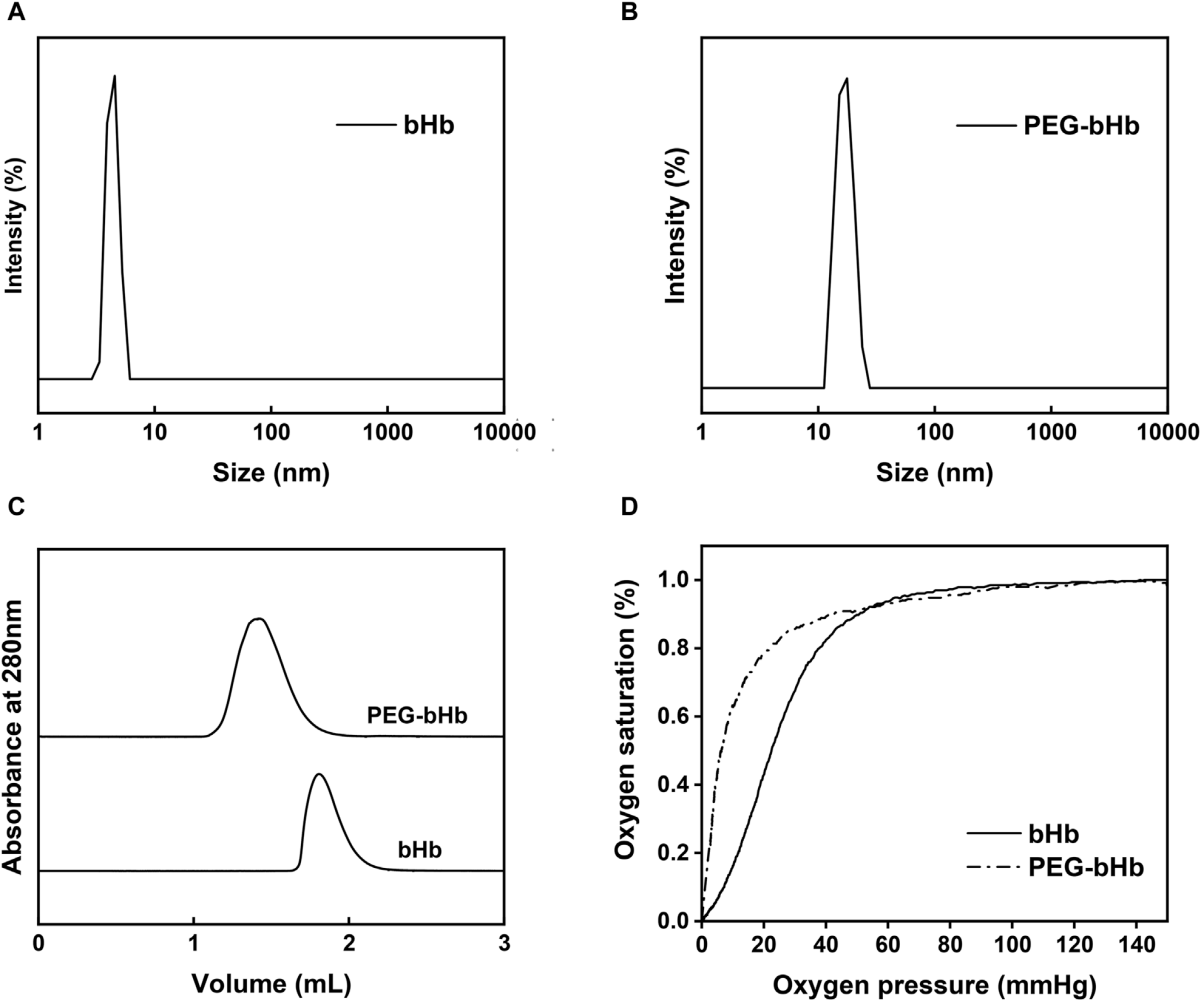
Characterization of bHb and PEG-bHb. **(A)** Hydration diameter distribution of bHb. **(B)** Hydration diameter distribution of PEG-bHb. **(C)** SEC curves of bHb and PEG-bHb. **(D)** Oxygen equilibrium curves of bHb and PEG-bHb.

PEG-bHb was synthesized and characterized. The synthetic route of PEG-bHb is shown in Supplementary Figure S1. The FTIR spectra of bHb and PEG-bHb are given in Supplementary Figure S2. The amide I peak of bHb was observed at 1,651 cm^-1^. A narrow absorption band at 1,089 cm^−1^ with high intensity corresponded to the C-O-C bond in PEG chains. The NMR spectra of bHb and PEG-bHb are given in Supplementary Figure S3. bHb exhibits multiple resonances in both the aliphatic (1.67 ppm) and aromatic (7.28 ppm) regions. A new chemical shift at 3.67 ppm appeared in the NMR spectrum of PEG-bHb, which belonged to all four protons of the ethylene oxide repeat unit of PEG. The hydration diameter distribution was analyzed using a dynamic light scattering analyzer. As shown in Figures 4A, B, both bHb and PEG-bHb had a single peak, indicating high purity. The hydration diameter of bHb was 4.7 ± 1 nm, while the hydration diameter of PEG-bHb increased to 15.5 ± 1.4 nm after PEGylation. Elution profiles of bHb and PEG-bHb were obtained. As shown in Figure 4C, bHb was eluted as a single peak at 1.78 mL, while PEG-bHb was eluted as a single and broad peak at 1.37 mL. Due to the PEG chain modification, the elution peak of PEG-bHb showed a significant left shift, with a broad peak reflecting its polydispersity.

To evaluate the oxygen-carrying and oxygen-releasing functions of bHb and PEG-bHb, the oxygen equilibrium curves were recorded and are shown in Figure 4D. The oxygen affinity of PEG-bHb was higher than that of bHb, as indicated by its lower P_50_ value (6.99 mmHg vs 23.73 mmHg), suggesting that PEGylation could improve the oxygen affinity.

Table 1 showed that PEG-bHb had a higher COP (64.1 mmHg) and viscosity (1.73 cp) than bHb (10.0 mmHg and 0.78 cp, respectively). This indicated that PEGylation could significantly increase the COP and viscosity of bHb.

**TABLE 1:** Characterization parameters.

Characterization parameter	bHb	PEG-bHb
^a^COP (mmHg)	10.0	64.1
^b^Viscosity (cp)	0.78	1.73
^c^P_50_ (mmHg)	23.73	6.99
Hydration diameter (nm)	4.7	15.5

COP, colloidal osmotic pressure; P_50_, partial pressure of oxygen when Hb oxygen saturation is 50%.

^a^ The COP of bHb and MalPEG-bHb was measured at a protein concentration of 40 mg/mL in PBS (pH 7.4).

^b^ The viscosity of bHb and MalPEG-bHb was measured at a protein concentration of 40 mg/mL with a shear rate of 75 s-1 at 37°C.

^c^ The P_50_ value of bHb and MalPEG-bHb was measured at a protein concentration of 0.75 mg/mL at 37°C.

### 4.4 Evaluation of PEG-bHb-induced vasoactivity

**FIGURE 5:**
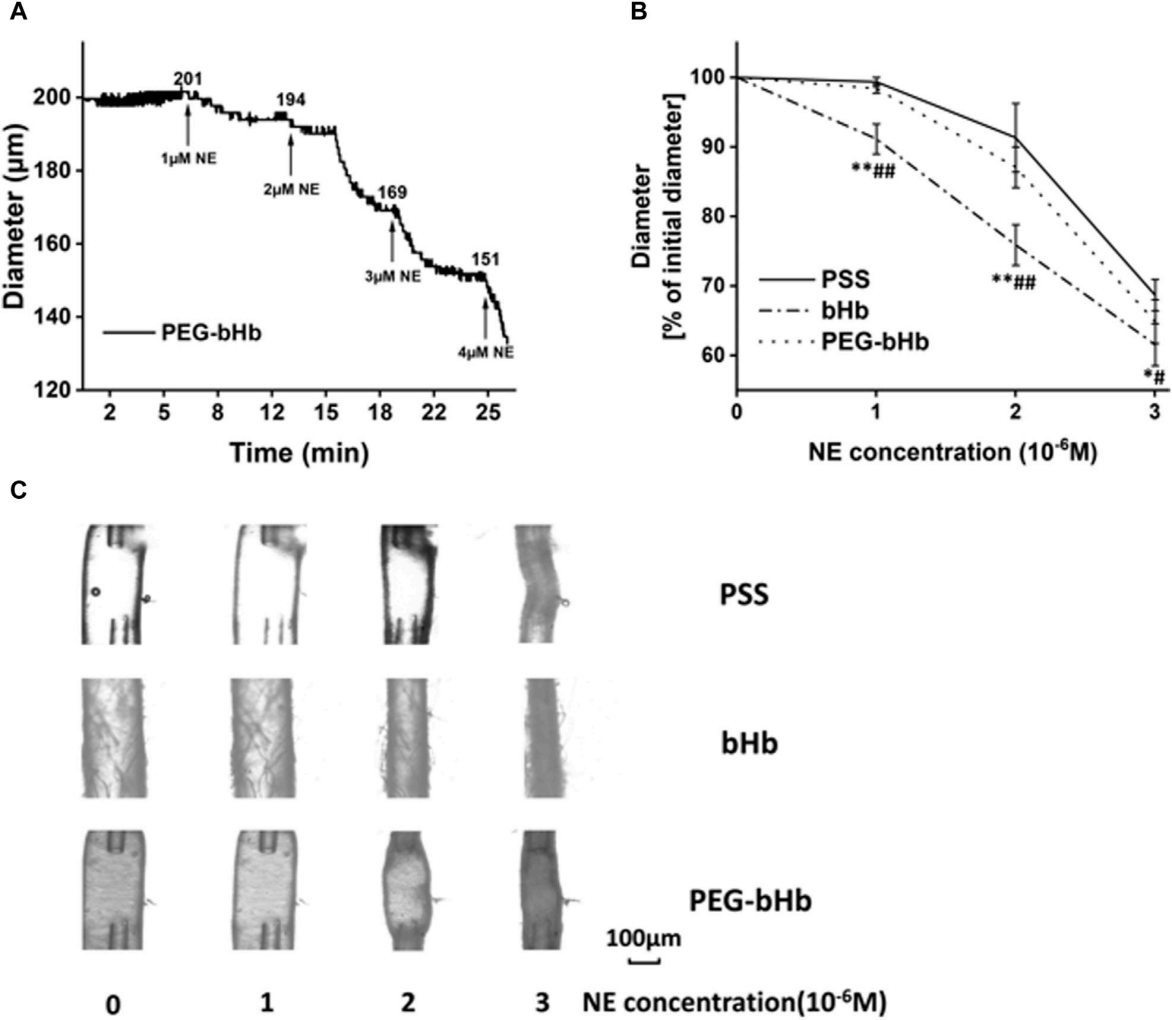
Response of vessels perfused with different samples for vasoactivity evaluation. **(A)** Diameter of the vessel perfused with PEG-bHb. **(B)** Percentage of the initial diameter of vessels perfused with PEG-bHb for vasoactivity evaluation (*n* = 3) (PSS buffer solution was considered the negative control, and bHb was considered the positive control; *P or ^#^ *p* < 0.05 and **P or ^##^ *p* < 0.01; *P: bHb vs PSS; ^#^P: bHb vs PEG-bHb). **(C)** Morphological images of vessels perfused with PSS, bHb, and PEG-bHb in the presence of NE (1 × 10^−6^∼3 × 10^−6^ M) in the bath.

The diameter change curve of the vessel after the perfusion of PEG-bHb was recorded and is shown in Figure 5A, and the percentage of vascular diameter constriction calculated from the initial value is shown in Figure 5B. When the concentration of NE increased from 1 × 10^−6^ M to 3 × 10^−6^ M, the vasoconstriction percentage of vessels perfused with PEG-bHb was 1.6%, 12.9%, and 35.1%. Compared with the PSS buffer solution, PEG-bHb induced slight vasoactivity. However, vasoactivity induced by PEG-bHb was significantly lower than that of bHb. Furthermore, PEG-bHb combined with CO (PEG-CObHb) was synthesized, and the vasoactivity induced by PEG-CObHb was evaluated (Supplementary Figure S4). Compared with bHb, the vasoconstriction percentage of vessels perfused with PEG-CObHb decreased with significant differences. Compared with PEG-bHb, the vasoconstriction percentage of vessels perfused with PEG-CObHb decreased slightly, but without a significant difference. As shown in Table 2, the coefficient of variation in the percentage of vasoconstriction was calculated to be less than 5%, indicating good reproducibility. The morphology of the vessels observed under the microscope after perfusion was photographed, and the images are shown in Figure 5C.

**TABLE 2:** Coefficient of variation.

Sample	PEG-bHb		
NE concentration (M)	1 × 10^−6^	2 × 10^−6^	3 × 10^−6^
***X (%***)	98.4	87.1	64.9
***SD (%***)	0.66	2.93	3.13
***CV (%***)	0.7	3.4	4.8

### 4.5 H&E staining of vessels after vasoactivity evaluation

**FIGURE 6:**
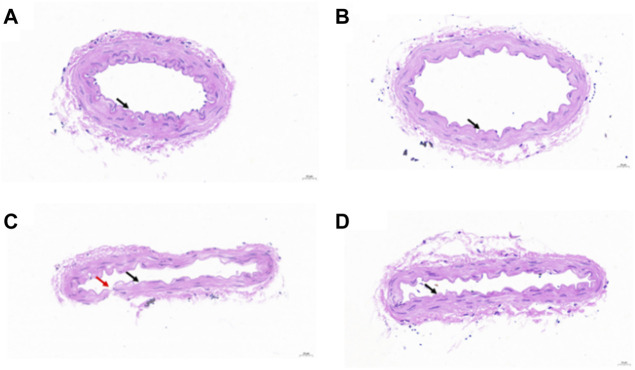
**(A)** H&E staining image of the native vessel. **(B)** H&E staining image of the vessel perfused with PSS. **(C)** H&E staining image of the vessel perfused with bHb. **(D)** H&E staining image of the vessel perfused with PEG-bHb.

The H&E staining image of native vessels is shown in Figure 6A. The results showed that the vascular wall structure was clear, the inner membrane was rarely missing endothelial cells (black arrow), the inner elastic plate was intact, and no obvious fracture was observed. The smooth muscle in the media was arranged regularly, the shape was normal, and the connective tissue in the outer membrane was arranged regularly. No obvious inflammatory cell infiltration was observed. The H&E staining image of the vessel perfused with PSS is shown in Figure 6B. The structure of the vascular wall was clear, the inner membrane was rarely missing endothelial cells (black arrow), and the inner elastic plate was occasionally broken (red arrow). The smooth muscle in the media was arranged regularly, the shape was normal, and the connective tissue in the outer membrane was arranged regularly. No obvious inflammatory cell infiltration was observed. The occasional fracture of the inner elastic plate may be due to physical damage caused by the tweezers during operation. The H&E staining images of the vessel perfused with bHb or PEG-bHb are shown in Figures 6C, D. The results indicated that the structure of the blood vessel wall was clear, the loss of endothelial cells in the intima was rare (black arrow), the inner elastic plate was intact, and no obvious fracture was observed. The smooth muscle in the media was arranged regularly, the shape was normal, and the connective tissue in the outer membrane was arranged regularly. No obvious inflammatory cell infiltration was observed. The H&E staining results showed that both bHb and PEG-bHb did not cause serious injury to the vascular endothelium.

## 5 Discussion

Vasoactivity induced by HBOCs was one of the major obstacles to its clinical application, which may cause gastrointestinal side effects, hypertension, chest pain, and even myocardial infarction (Zhang et al., 2016). The establishment of a vasoactivity evaluation method is of great significance for the development of HBOCs, especially for the study of vasoactivity induction mechanisms and inhibition methods. In this study, an *ex vivo* method for evaluating vasoactivity induced by HBOCs was established based on resistance vessels and commercial devices.

The DMT120CP system is a commercial device that simulates physiological conditions for isolated vessels and measures changes in vessel diameter. According to literature reports, the DMT120CP system is usually applied for monitoring the effects of small molecules on isolated vessels (Enouri et al., 2011; Schjorring et al., 2012; Tian et al., 2012). The small molecules are added directly to the buffer solution in the bath, pass through the blood vessel wall, and act on blood vessels. After the physical encapsulation or chemical modification, HBOCs presented a particle size of more than 5 nm usually, and it is difficult to penetrate the vessel wall. To solve this problem, HBOCs were perfused into the vessel directly. During the experiments, the vessel was equilibrated with PSS buffer solution prior to HBOC perfusion. The exchange of the perfusate from PSS to HBOCs caused bubbles to form in the vessel, interfering with the measurement of the vascular diameter and leading to experiment failure. To solve this problem, a micro-injection pump and a three-way valve device were installed at the entrance of the vessel. Before exchanging the perfusate from PSS to HBOCs, the entire flow path was filled with HBOCs to expel the air. The three-way valve was then moved to connect the flow path to the entrance of the vessel. After verification, the modified DMT120CP system was successfully used for evaluating vasoactivity induced by HBOCs. To the best our knowledge, this is the first time that the DMT120CP system was applied for the measurement of nanoscale samples.

Resistance vessels, which are rich in smooth muscle, play a crucial role in regulating vessel diameter and maintaining normal blood pressure (Guan et al., 2018; Quek et al., 2018; Horn et al., 2019). Given their importance in blood pressure regulation, rat mesenteric resistance vessels were used for *ex vivo* vasoactivity evaluation. Gisolfi C. V et al. previously isolated mesenteric artery segments from male SD rats (250–300 g) with a diameter ranging from 100 to 340 μm (Looft-Wilson et al., 1999). In this study, the mesenteric artery of male SD rats was isolated and a length of 2 mm was cut. The diameter of the isolated rat mesenteric artery ranged from 110 μm to 160 μm before reaching equilibrium and was consistent with previous literature reports (Looft-Wilson et al., 1999; Michea et al., 2005).

After reaching equilibrium, the diameter of the isolated rat mesenteric arteries increased significantly, ranging from 150 μm to 200 μm (Schjorring et al., 2012). By adjusting the pressure at both ends of the vessel to simulate the physiological environment in the body, the diameter of the mesenteric artery was increased. In this study, HBOC-induced vasoactivity based on the mesenteric artery *ex vivo* was measured using the DMT120CP system and characterized by diameter changes.

Free bHb is prone to extravasation from the vascular lumen and can bind to the relaxation factor NO released by endothelial cells, which can induce vasoactivity (Riess, 2001; Portoro et al., 2020; Song et al., 2020). Therefore, perfusion of bHb into the vessel was set as the positive control, while the PSS solution was used as the negative control. Compared to that in the negative control group, the vasoconstriction was not significant after the perfusion of bHb. The experimental result was consistent with that reported by Xiong Y. et al., in which Hb did not significantly affect the arteriolar tone during the perfusion, and then the vasoconstrictor angiotensin II was introduced to measure the effect of free Hb solution on the afferent arteriole diameter. Compared with the control solution, Hb significantly increased the response of afferent arterioles to angiotensin II. NE, another vasoconstrictor, was used as an adjuvant to supplement the blood volume and increase the blood pressure in this study. It has been reported that the vasoconstriction induced by NE is dose-dependent, and with the decrease in NO bioavailability, the vasoconstriction induced by NE is enhanced (Nishida et al., 1998; Lembo et al., 2000). Moreover, the experimental protocols ensured that endothelial cell integrity was maintained in the isolated blood vessel during equilibrium, thereby preserving endothelium-derived NO productivity (Enouri et al., 2011). In this study, NE was introduced to measure the effect of the bHb solution on the vessel diameter of the mesenteric artery. It was found that the vascular diameter changes in bHb and PSS perfusion showed a significant difference with the NE concentration ranging from 1 × 10^−6^ to 3 × 10^-6^ M. Bhb-induced vasoconstriction was significantly enhanced. Therefore, based on the DMT120CP system and isolated mesenteric artery vessels, NE was introduced to measure the influence of HBOC samples on the vascular diameter.

Compared with free Hb, the vasoactivity induced by HBOCs was significantly reduced by increasing the molecular diameter via liposome encapsulation, cross-linking, polymerization, or polymer modification. MP4 (Sangart, United States), mPEG-Mal-modified human Hb, has been shown to provide oxygen to hypoxic tissue effectively and completed phase I and II clinical trials. According to the literature report, there was no significant elevation in MAP during or after exchange transfusion of MP4 in rats (Vandegriff et al., 2003), while according to reports of major adverse reactions in clinical trials, when MP4 was used to treat hemorrhagic shock, the incidence of hypertension in the experimental group was higher than that in the control group (Natanson, 2008; Liu and Yang, 2022). In this study, the observed changes in the characterization parameters of PEG-bHb were in line with those reported in the literature, providing evidence for the successful modification of PEG chains (Vandegriff et al., 2003; Wang et al., 2017). The modification of mPEG-Mal effectively increased the molecular diameter and weight of bHb, potentially hindering the extravasation of the vascular wall, reducing the scavenging effect of NO molecules, and ultimately achieving the goal of reducing vasoactivity. Additionally, the isolated vessel was in a high-oxygen, partial-pressure environment for maintaining endothelial integrity. It cannot be concluded whether the strong oxygen affinity based on PEG-bHb plays a role in reducing vasoactivity. The vessel perfused with PEG-bHb was used as the experimental group for vasoactivity evaluation, with samples synthesized in different batches evaluated to determine the repeatability of the results. The vasoconstriction percentage coefficient of variation was less than 5%, indicating good reproducibility. Compared with bHb perfusion, the vessel perfused with PEG-bHb exhibited significantly reduced vasoconstriction, which was consistent with previously reported results (Vandegriff et al., 2003; Wang et al., 2017). However, compared with PSS perfusion, the vessel perfused with PEG-bHb exhibited increased vasoconstriction, indicating minor vasoactivity.

In conclusion, we established an *ex vivo* method for evaluating HBOC-induced vasoactivity based on the modified DMT120CP system and mesenteric artery resistance vessels. We optimized the NE concentration to 1 × 10^−6^∼3 × 10^−6^ M as the vasoactivity magnifier and perfused PEG-bHb, bHb, and PSS into the vessel as the test sample, positive control, and negative control, respectively. However, further evaluation is needed to assess vasoactivity induced by other types of HBOCs and verify the stability and universality of this method. Additionally, future modifications can be made by comparing the results obtained using this method with those obtained by clinical trials or *in vivo* studies in order to improve accuracy. In summary, this study provides a simple and rapid method for evaluating HBOC-induced vasoactivity, which can be used to investigate mechanisms, eliminate vasoactivity, and ultimately promote the clinical use of HBOCs.

## Data Availability

The original contributions presented in the study are included in the article/Supplementary Material; further inquiries can be directed to the corresponding authors.
