# Supplementary material for: An ex vivo method to evaluate vasoactivity induced by hemoglobin-based oxygen carriers in resistance vessels
Source: Front Bioeng Biotechnol. 2024 Jun 28;12:1376806. doi: 10.3389/fbioe.2024.1376806 (PMC11239391; doi:10.3389/fbioe.2024.1376806)
Supplement: Supplementary file 1 [file DataSheet1.PDF]

Supplementary Material

An ex vivo method may evaluate vasoactivity induced by Hemoglobin-Based Oxygen Carriers in resistance vessels

Hang Yu[1]†, Daoyuan Gao[1]†, Guoxing You[1], Weidan Li[1], Ying Wang[1], Yuzhi Chen[1]*, Lian Zhao[1]*

*** Correspondence:**

Corresponding Author: Yuzhi Chen, Lian Zhao

chenyuzhi_buct@163.com (Yuzhi Chen)

zhaolian@bmi.ac.cn (Lian Zhao)

1 Supplementary Figures

Supplementary Figure S1. Synthetic route of PEG-bHb

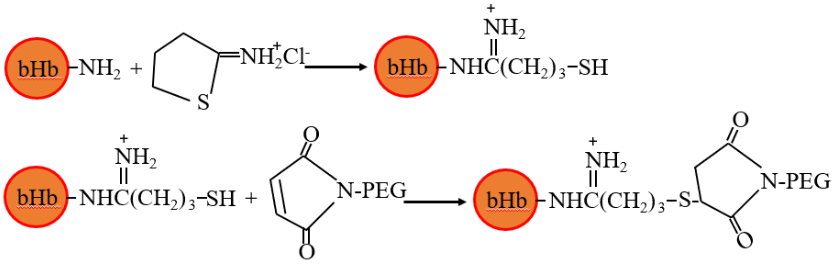

Figure S1. Schematic representation of the reactions to produce PEG-bHb.

Supplementary Figure S2. FTIR spectra of bHb and PEG-bHb

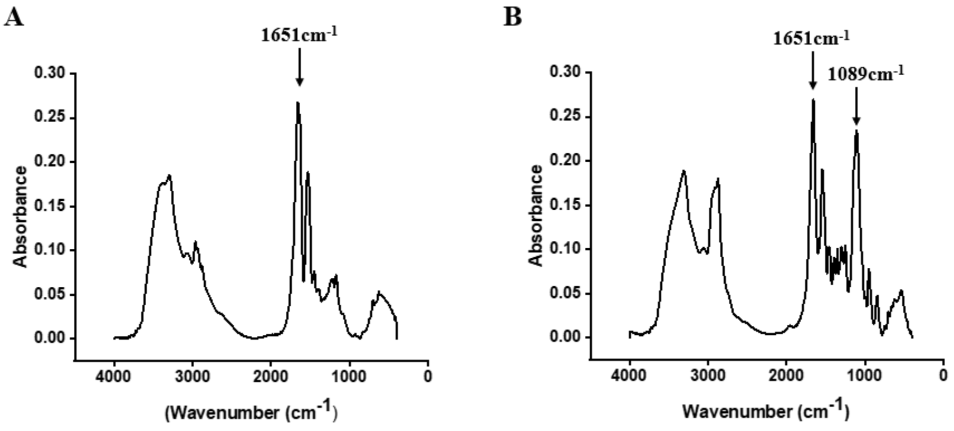

Figure S2. (A) FTIR spectrum of bHb. (B) FTIR spectrum of PEG-bHb.

Supplementary Figure S3. NMR spectra of bHb and PEG-bHb

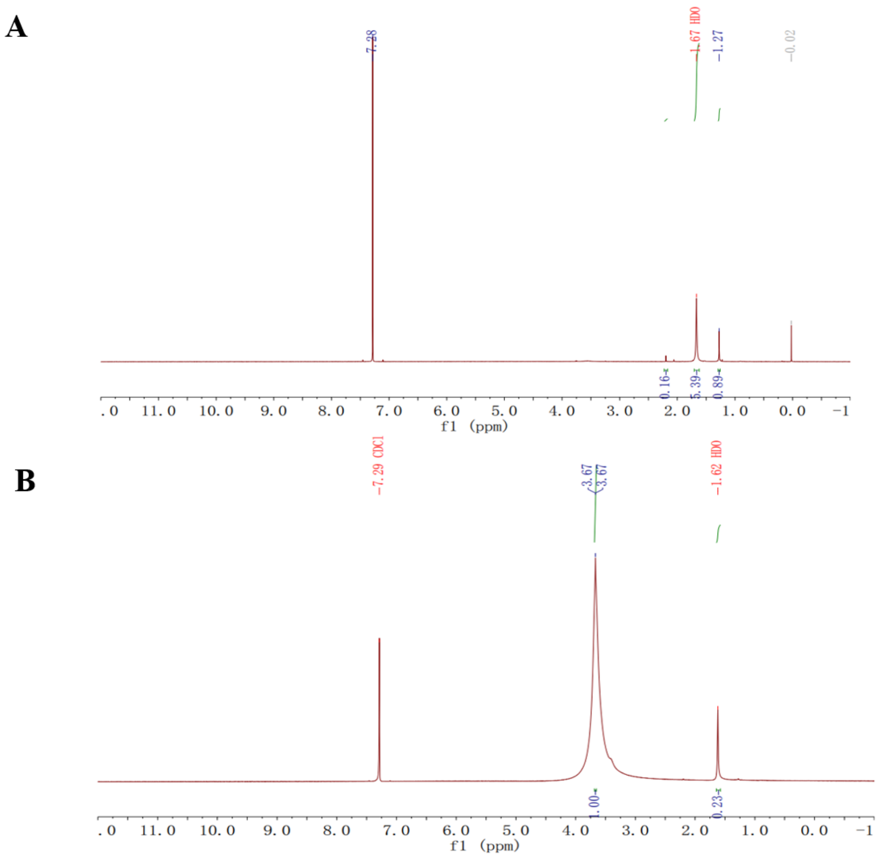

Figure S3. (A) NMR spectrum of bHb. (B) NMR spectrum of PEG-bHb

Supplementary Figure S4. Evaluation of vasoactivity induced by PEG-CObHb

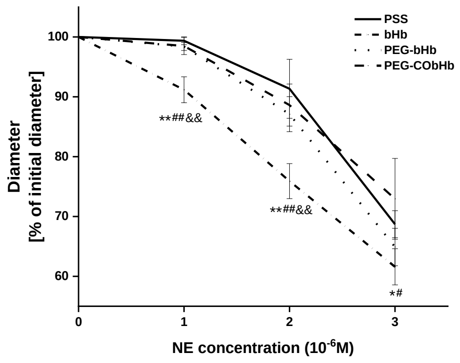

Figure S4. Percentage of the initial diameter from vessels perfused with PEG-bHb for vasoactivity evaluation (n=4) (PSS buffer solution as the negative control, bHb as the positive control; *P, #P or &P < 0.05 and **P , ##P or &&P < 0.01; *P: bHb vs PSS, #P: bHb vsPEG-bHb,&P:bHbvsPEG-CObHb).
